# Impact of high-altitude exposure on cerebral lobe functions in climbers: insights from the Nepali Himalayas

**DOI:** 10.3389/fnsys.2025.1563398

**Published:** 2025-05-30

**Authors:** Sunil Dhungel, Shavana R. L. Rana, Arun Kumar Neopane, Barun Mahat, Bipin Kumar Shrestha, Yesha Shree Rajaure, Bikalp Thapa, Naveen Phuyal, Naresh Manandhar, Udaya Shrestha, Suraj Parajuli, Taraman Amatya

**Affiliations:** ^1^Department of Clinical Physiology, College of Medicine, Nepalese Army Institute of Health Sciences, Kathmandu, Nepal; ^2^Department of Neuroscience, Medical University of the Americas, Charlestown, Saint Kitts and Nevis; ^3^Department of Pediatrics, Shree Birendra Hospital, College of Medicine, Nepalese Army Institute of Health Sciences, Kathmandu, Nepal; ^4^Department of Community Medicine, College of Medicine, Nepalese Army Institute of Health Sciences, Kathmandu, Nepal; ^5^Shree Birendra Military Hospital (SBH), Kathmandu, Nepal; ^6^Department of Clinical Skills, Medical University of the Americas, Charlestown, Saint Kitts and Nevis

**Keywords:** cerebral lobe function, acclimatation, Nepal, hypoxia, high-altitude

## Abstract

**Introduction:**

High-altitude environments challenge cognitive function due to hypoxia, yet their specific effects on cerebral lobe functions remain unclear. This study examines the impact of high-altitude exposure on frontal, parietal, temporal, and occipital lobes in climbers in the Nepali Himalayas, aiming to enhance understanding of altitude-related cognitive decline.

**Methods:**

A cross-sectional cohort study was conducted with 76 participants, including 46 non-selected individuals (NOSCL) and 30 selected climbers divided into Everest (EMCL, *n* = 12), Kanchanjanga (KMCL, *n* = 9), and Manaslu (MMCL, *n* = 9) groups. Cognitive function tests (CFT) assessed cerebral lobe function at altitudes ranging from 800 to 5,500 meters using a non-invasive neuropsychological battery.

**Results:**

Significant altitude-related declines were observed in frontal lobe function, particularly in the Visual Stroop test at 800 meters (75%, *p* < 0.001) and 2,700 meters (86.1%, *p* < 0.001). Attention scores also decreased at 800 meters (94.4%, *p* = 0.002). No significant changes were found in parietal, temporal, or occipital lobe functions. The Manaslu climb presented greater cognitive challenges than Everest or Kanchanjanga, with reduced attention and social cognition scores at 4,800 meters (*p* = 0.145).

**Discussion:**

The findings indicate that frontal lobe functions are particularly vulnerable to hypoxia at high altitudes. The results support the necessity of region-specific cognitive testing for high-altitude risk assessments. Further research should explore long-term cognitive effects and mitigation strategies for climbers exposed to extreme altitude conditions.

## 1 Introduction

High-altitude environments, such as the Nepali Himalayas, present unique physiological and cognitive challenges due to reduced oxygen levels. Over 140 million people live at altitudes above 3,000 meters, with the Himalayas spanning approximately 1,000,000 square kilometers across Asia (Cumpstey et al., 2020; Inside Himalayas, n.d.). Nepal, home to eight of the world’s fourteen highest peaks, including Mount Everest (8,848 m), has 42% of its landmass classified as high-altitude regions (Tamrakar, 2003). High-altitude sickness, which occurs between 1,500 and 3,500 meters, encompasses a spectrum of conditions ranging from acute mountain sickness (AMS) to life-threatening high-altitude pulmonary edema (HAPE) and high-altitude cerebral edema (HACE) (Sharma et al., 2019; Bärtsch and Swenson, 2013; Marmura and Hernandez, 2015).

High-altitude hypoxia primarily affects the nervous system, leading to cognitive impairments such as memory loss, attention deficits, and executive dysfunction (Bärtsch and Swenson, 2013; Marmura and Hernandez, 2015; Linde et al., 2017). These effects are more pronounced in unacclimatized lowlanders, though some impairments may persist even after returning to low altitudes (West, 1986). While previous studies have explored general cognitive decline at high altitudes, there is limited research on region-specific cognitive impairments, particularly in climbers exposed to extreme altitudes. This study aims to address this gap by examining the effects of high-altitude exposure on cerebral lobe functions, focusing on the frontal, parietal, temporal, and occipital lobes.

The rationale for this study lies in its potential to develop strategies for mitigating cognitive risks in high-altitude environments. By identifying specific brain regions affected by hypoxia, this research contributes to the development of protocols for climbers and high-altitude workers. The study builds on previous research by using non-invasive neuropsychological batteries to assess region-specific cognitive impairments, providing a comprehensive understanding of how high altitudes impact brain function.

## 2 Materials and methods

### 2.1 Study design

This cross-sectional cohort study assessed cerebral lobe functions (CFT) at various altitudes in Nepal, including Pokhara (800 meters), Jomsom (2,700 meters), Kaisang (3,500 meters), and Muktinath (3,800 meters). Additional assessments were conducted at Everest Base Camp (EBC), Kanchanjanga Base Camp (KBC), and Manaslu Base Camp (MBC). The study was part of the “Safa Himal Aviyan” project, a mountain cleanup campaign led by the Nepalese Army (NA).

### 2.2 Participants

Participants were regular military troops recruited for the Mountain Cleanup Campaign 2022 (Headline Nepal, 2022). A total of 76 participants were initially enrolled, with 46 meeting the common baseline qualifying criteria (e.g., normal BMI, no history of head trauma, abstinence from alcohol/drugs) but not selected for climbing. The remaining 30 participants, who met both common and special qualifying criteria (e.g., low-altitude birth, non-Sherpa ethnicity), were divided at High altitudes mountain warfare School located in Jomsom (2,700 meters, Mustang district, Nepal) into three groups: Everest Mountain Climb (EMCL, *n* = 12), Kanchanjanga Mountain Climb (KMCL, *n* = 9), and Manaslu Mountain Climb (MMCL, *n* = 9).

### 2.3 Procedures

Participants underwent a general medical examination and Mini-Mental State Examination (MMSE) at Shree Birendra Hospital, Kathmandu, before CFT assessments. Acclimatization periods ranged from three to seven nights at each altitude station. CFT assessments were conducted using a structured proforma, with trained medical professionals administering and scoring the tests. Questionnaires-base working CFT proforma was designed (Blumenfeld, 2010a; Montreal Cognitive Assessment, n.d.; Kolb and Whishaw, 2009; Jones et al., 2012; Kaplan and Sadock, 2015; Blumenfeld, 2010b), adding a Nepali language subheading as well. The acclimatization period of 3–7 nights at each altitude station was maintained.

### 2.4 Neuropsychological batteries

The CFT proforma included four sections:

Frontal lobe examination (FLE): Eleven test batteries assessing attention, social cognition, abstract reasoning, and response suppression.
Parietal lobe examination (PLE): Ten test batteries evaluating spatial awareness, calculations, and apraxia.
Temporal lobe examination (TLE): Five test batteries for sound perception, auditory speech recognition, and face identification.
Occipital lobe examination (OLE): Two test batteries for visual field and color perception.

Each participant was given 20–30 min to read the CFT proforma and was explained which tests the participants should perform on their own and which tests would be performed by medical professionals. The worksheet also included written instructions for each battery of tests, as well as any necessary illustrations. Again, a time of 30 to 45 min was given to each participant to finish the test. The allocated trained doctors had to score the test as it was being administered using a separate checklist based upon “Circle appropriately 0-1-2,” the scoring system (0-wrong, 1-half correct, 2-correct). To avoid inter and intra-observer bias, the data collection crew (medical doctors of NA) had been trained for 3 h per day for 3 days on the questionnaire. Testers were blinded to participant altitude exposure groups during assessments.

### 2.5 Ethical considerations

The study protocol was approved by the Institutional Review Committee of the Nepalese Army Institute of Health Sciences (IRC-NAIHS; Reg. No. 597, February 2022). All procedures adhered to the Declaration of Helsinki and Nepal Health Research Council (NHRC) guidelines, with informed consent obtained from all participants.

### 2.6 Statistical analysis

#### 2.6.1 *A priori* power analysis

*A priori* power analysis was conducted using G Power software (version 3.1) to determine the required sample size. Based on an expected medium effect size (*f* = 0.25), *α* = 0.05, and power (1 − *β*) = 0.80, the analysis indicated a minimum sample size of 66 participants for a repeated-measures ANOVA with three groups and four measurements. The final sample size of 76 participants exceeded this requirement, ensuring adequate statistical power. A value for probability less than 0.05 (*p* < 0.05) at a 95% confidence interval is considered statistically significant. In data analysis, we calculate the percentage of total scores (based on responses) by dividing the total scores from each set of questionnaires in the test batteries by 100. Data normality was assessed using the Shapiro–Wilk test. For parametric data, a repeated-measures ANOVA was employed to compare means across multiple time points and groups, with altitude and group as within- and between-subjects factors, respectively. For non-parametric data, the Friedman test was used, followed by *post-hoc* Wilcoxon signed-rank tests with Bonferroni correction. Effect sizes were calculated using partial eta-squared (*η*^2^) for ANOVA and Cohen’s *d* for *post-hoc* comparisons. These measures were reported to quantify the magnitude of observed effects. To account for multiple comparisons, Bonferroni correction was applied to all *post-hoc* tests. Pairwise comparisons were conducted to identify specific differences between groups and altitudes. Sex was included as a fixed factor in a two-way ANOVA to examine potential interactions between sex and altitude on cognitive performance. Additionally, a regression model was used to assess the influence of sex on frontal lobe function scores.

## 3 Results

**Figure 1:**
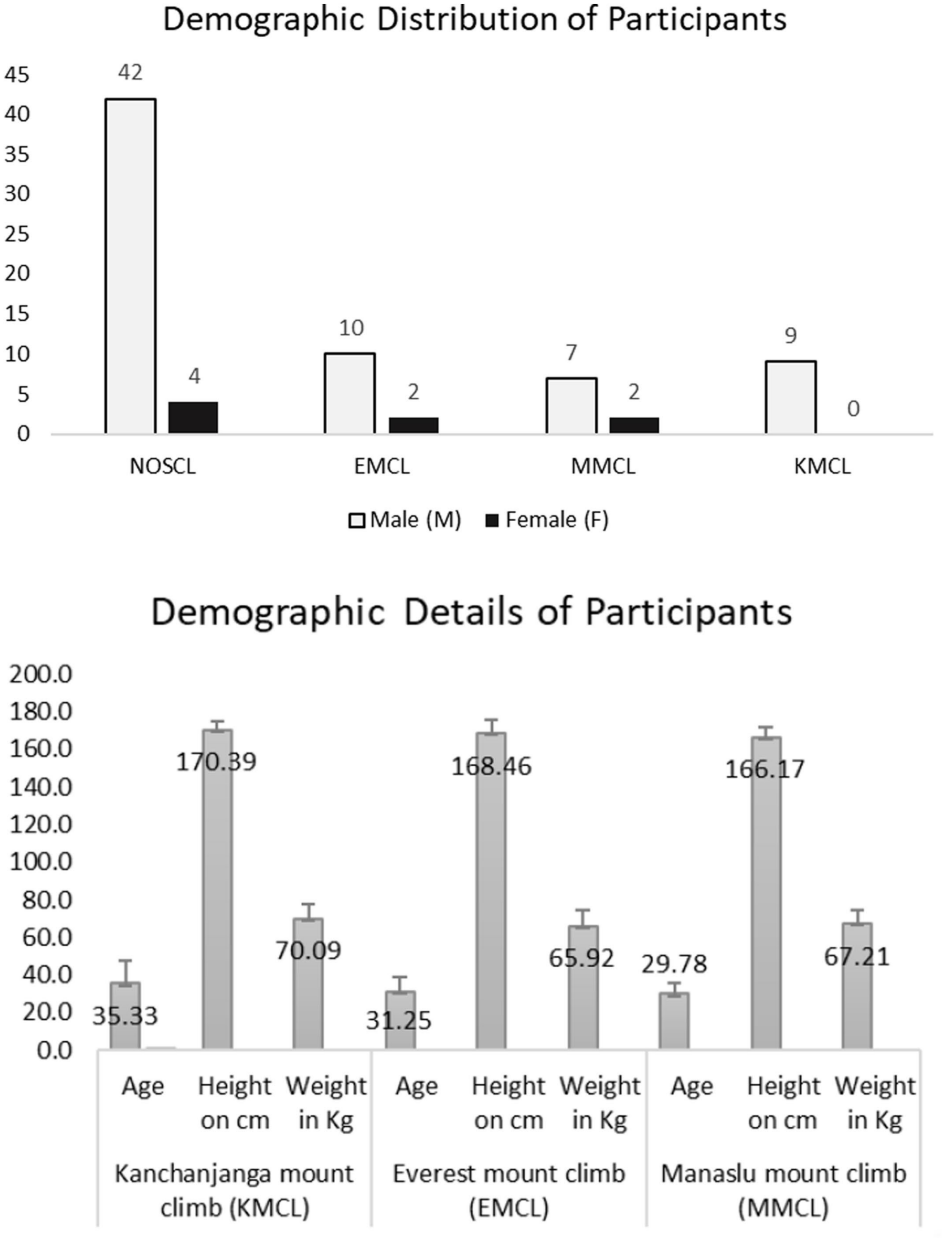
Demographic distribution of participants. NOSCL, enrolled but not-selected participants at Jomsom; EMCL, Everest Mountain Climb at Everest Base Camp; MMCL, Manaslu Mountain Climb at Manaslu Base Camp; KMCL, Kanchanjanga Mountain Climb at Kanchanjanga Base Camp. The number above each bar represents the gender count of participants in demographic distributions of participant’s bar diagram and data are presented as mean ± standard deviation in demographic details of participant’s bar diagram.

**Figure 2:**
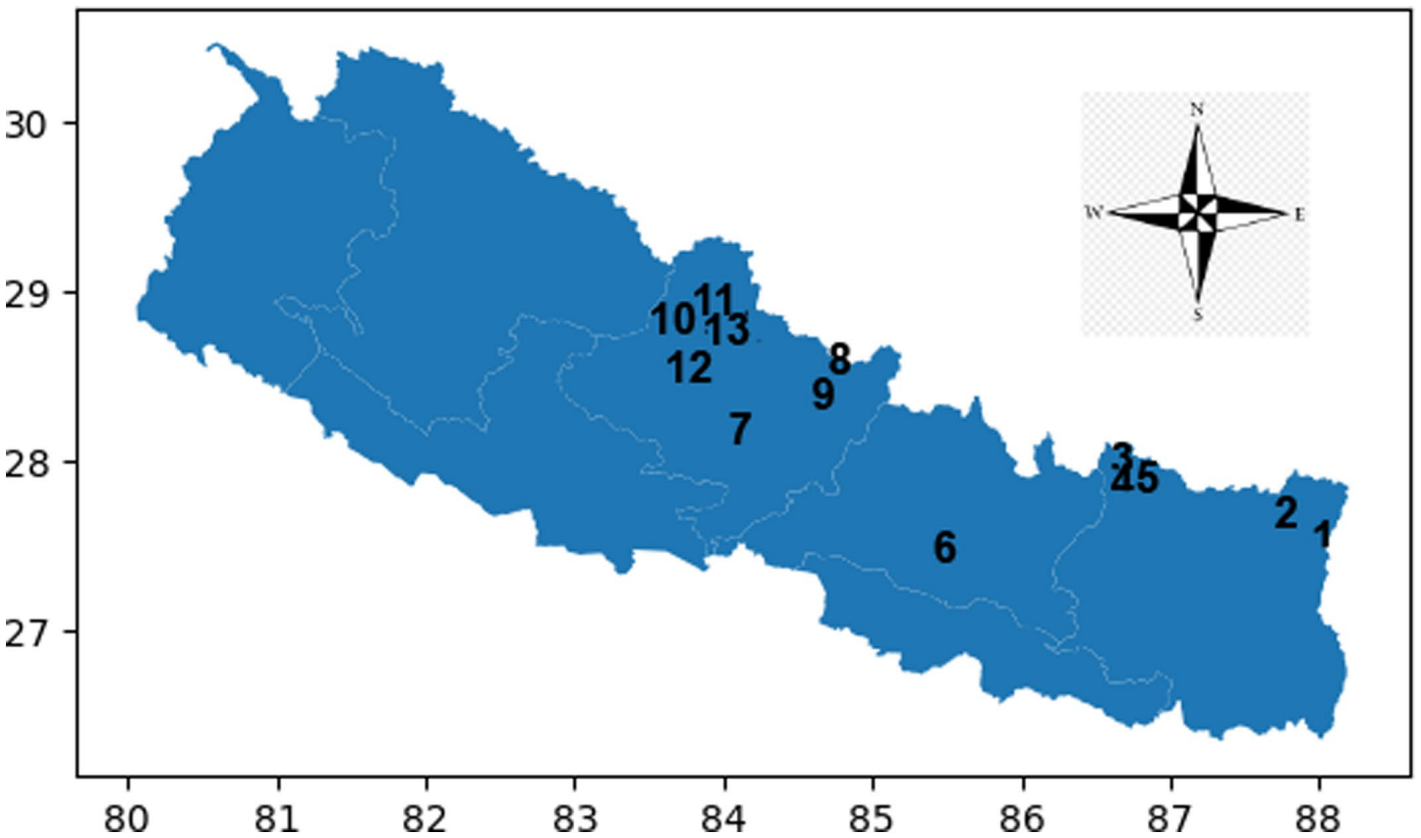
Map illustrating the altitude stations and the CFT recordings at each station. Nepal latitudes and longitudes map Y-axis north and X-axis east. Numbers seen in the map are CFT recording stations at different altitude stations. Number 6 & 7 indicate low altitude and the rest are high altitude stations. *CFT recording stations of Kanchanjanga Base Camp climbers (KBCL)* 6: Kathmandu (1,400 meter); 7: Pokhara (800 meter); 12: Jomsom (2,700 meter); 13: Kaisang (3,500 meter); 10: Muktinath (3,800 meter) 1: Tseram 3,870 meter, 2: Kanchanjanga Base Camp (5,475 meter); 11: Thorang (5,400 meter). *CFT recording stations of Everest Base Camp climbers (EBCL)* 6: Kathmandu (1,400 meter); 7: Pokhara (800 meter); 12: Jomsom (2,700 meter); 4: Namche (3,400 meter); 13: Kaisang (3,500 meter); 10: Muktinath (3,800 meter); 5: Dengboche (4,400 meter); 11: Thorang (5,400 meter); 3: Everest Base Camp (5,500 meter). *CFT recording stations of Manaslu Base Camp climbers (MBCL)* 6: Kathmandu (1,400 meter); 7: Pokhara (800 meter); 12: Jomsom (2,700 meter); 13: Kaisang (3,500 meter); 10: Muktinath (3,800 meter); 9: Manaslu Base Camp (4,800 meter); 8: Samagau (4,900 meter).

**Figure 3:**
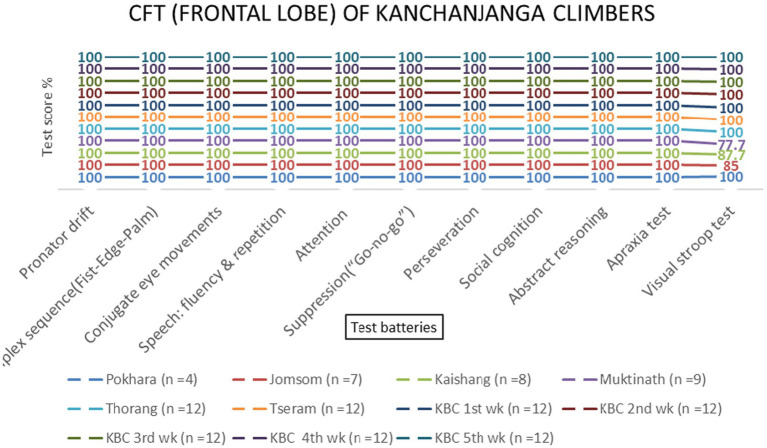
Frontal lobe examination scores of KMCL group across altitude stations (including once per week total four times at Kanchanjanga Base Camp) based on 11 test batteries.

Key results are summarized below, with detailed statistical values and CFT test score sheet and proforma are included in the Supplementary material (see Figures 1–3).

### 3.1 Frontal lobe functions

**Figure 4:**
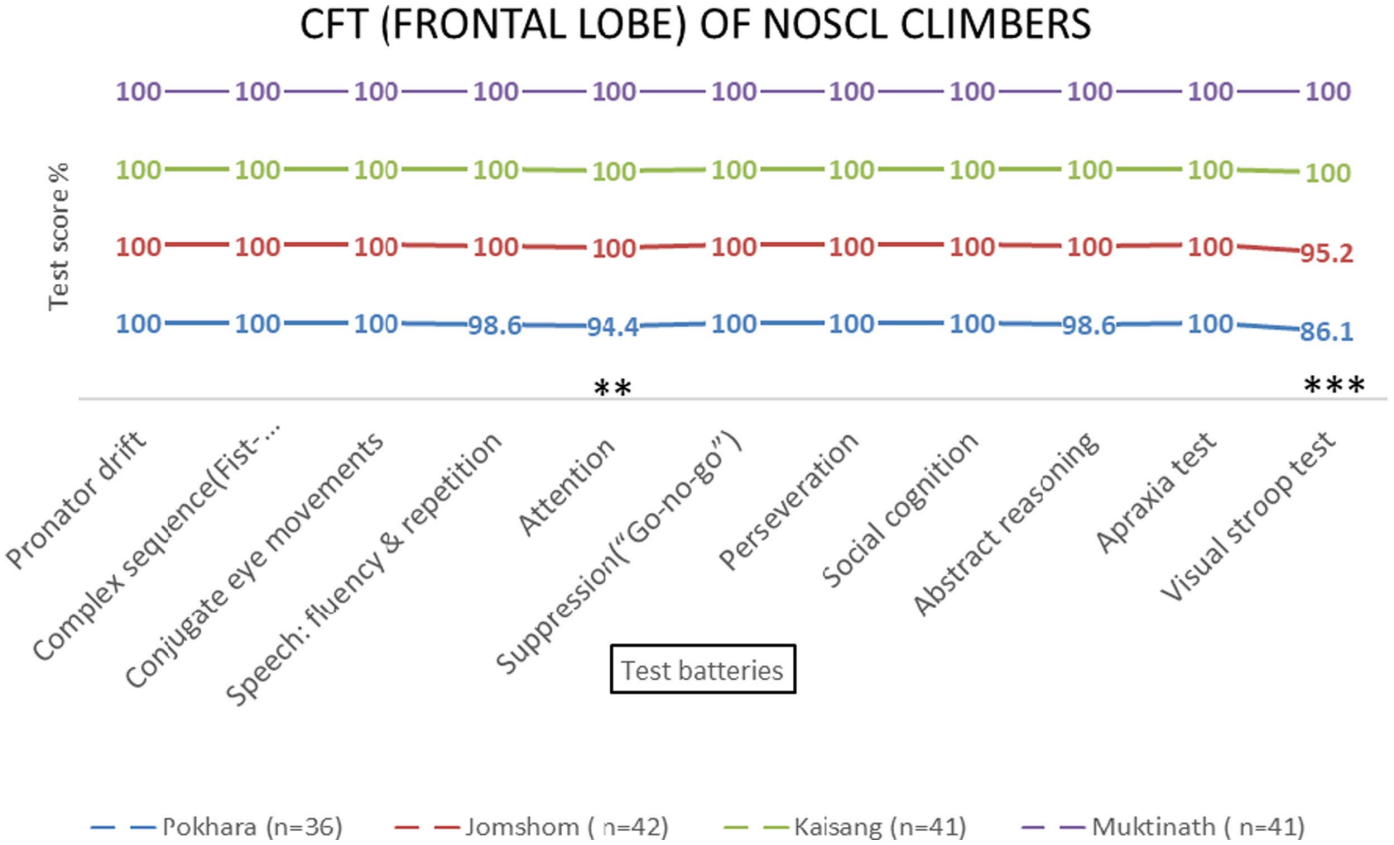
Frontal lobe examination scores of NOSCL group across four altitude stations based on 11 test batteries. Significant differences (^**^p < 0.01 and ^***^p < 0.001) are indicated.

**Figure 5:**
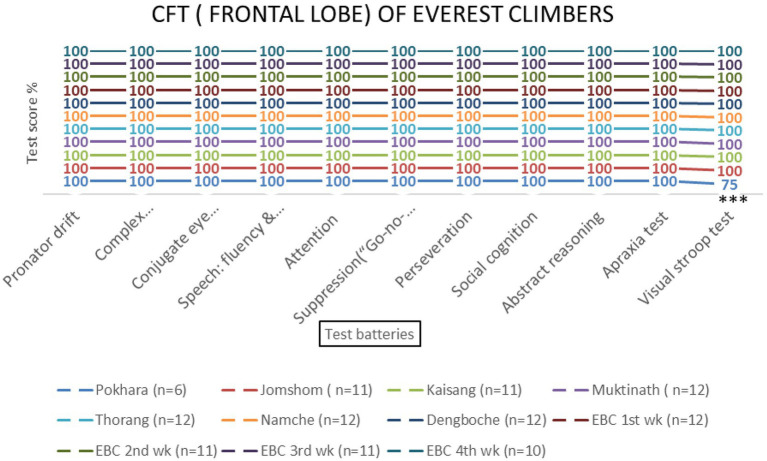
Frontal lobe examination scores of EMCL group at 11 altitude stations (including once per week total four times at Everest Base Camp) based on 11 test batteries. Significant differences (^***^p < 0.001) is indicated.

Repeated-measures ANOVA revealed significant effects of altitude on frontal lobe functions [*F* (3, 69) = 8.45, *p* < 0.001, *η*^2^ = 0.27]. *Post-hoc* tests indicated that the Visual Stroop test scores decreased significantly at 800 meters (75%, *p* < 0.001) and 2,700 meters (86.1%, *p* < 0.001). Attention scores also declined at 800 meters (94.4%, *p* = 0.002) and 4,800 meters (87.5%, *p* = 0.145) (Table 1 and Figures 4, 5). No significant sex-based differences were observed [*F* (1, 74) = 0.56, *p* = 0.456].

**Table 1:** ANOVA results for frontal lobe functions.

Test battery	*F*-value	*p*-value	Partial *η*^2^	*Post-hoc* (Bonferroni)
Visual Stroop	8.45	<0.001	0.27	800 m < 2,700 m (*p* < 0.001)
Attention	5.67	0.002	0.18	800 m < 4,800 m (*p* = 0.002)
Social cognition	3.12	0.145	0.10	NS

### 3.2 Parietal lobe functions

**Figure 6:**
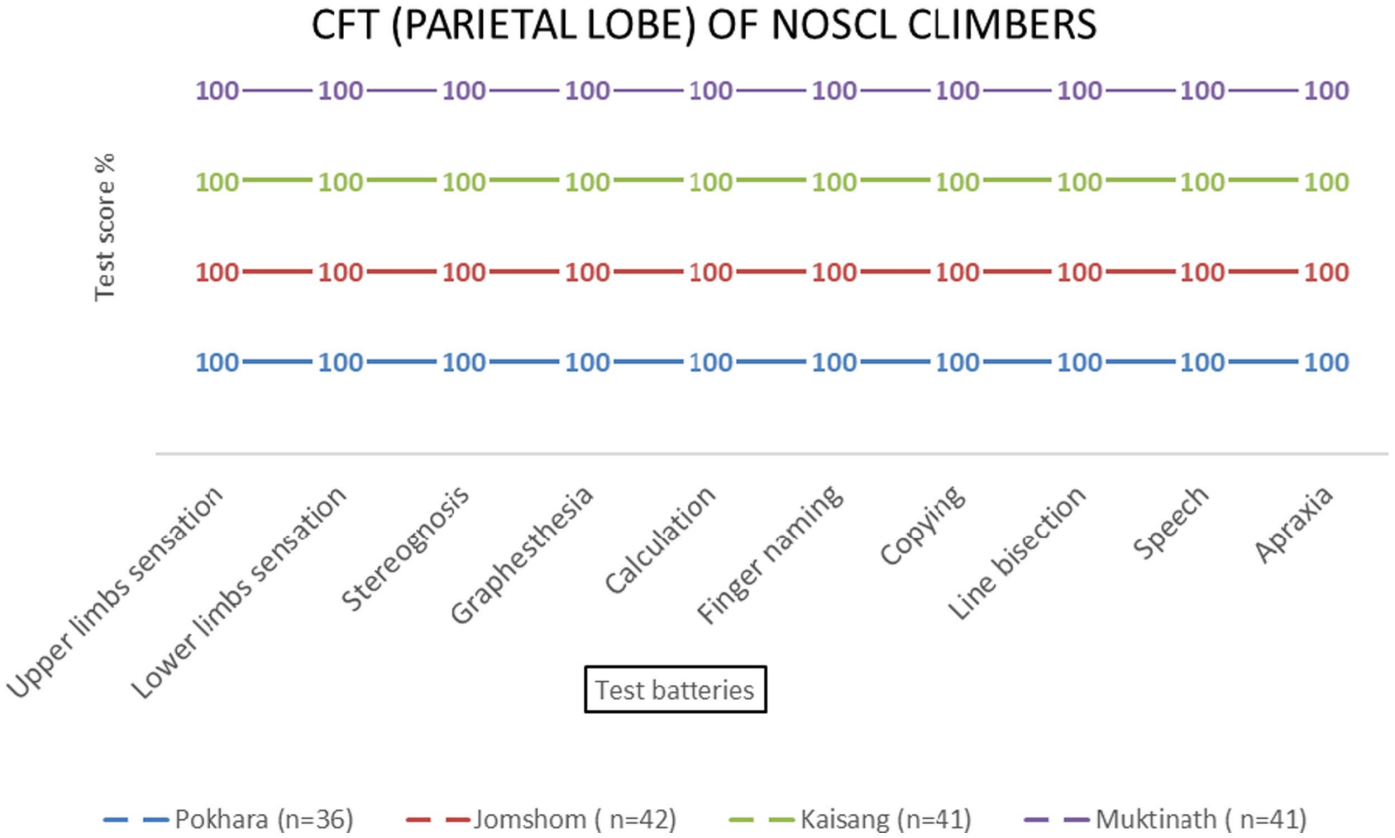
Parietal lobe examination scores of NOSCL group at four stations based on 10 test batteries.

**Figure 7:**
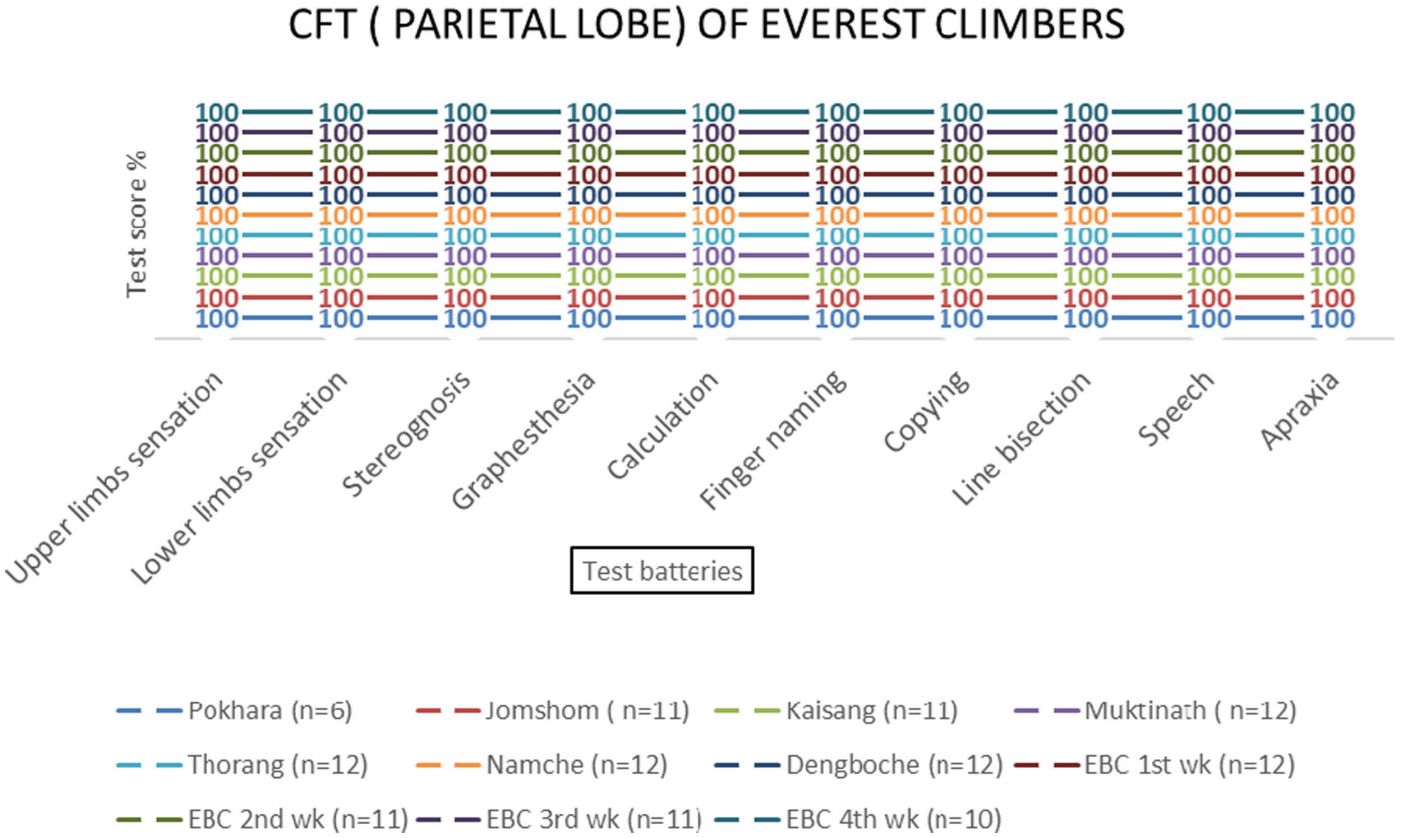
Parietal lobe examination scores of EMCL group at 11 altitude stations (including once per week total four times at Everest Base Camp) based on 10 test batteries.

**Figure 8:**
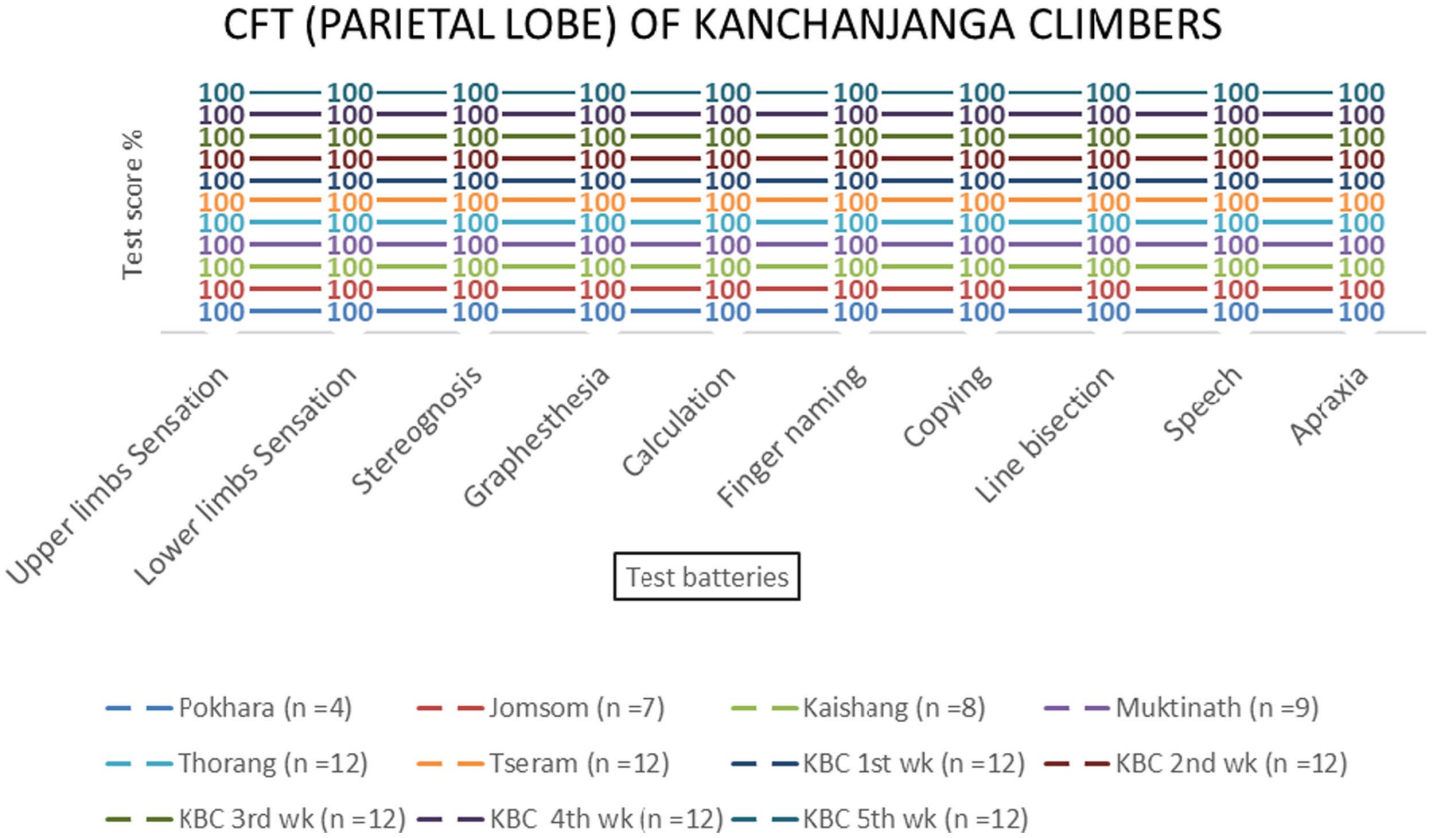
Parietal lobe examination scores of KMCL group across altitude stations (including once per week total four times at Kanchanjanga Base Camp) based on 10 test batteries.

**Figure 9:**
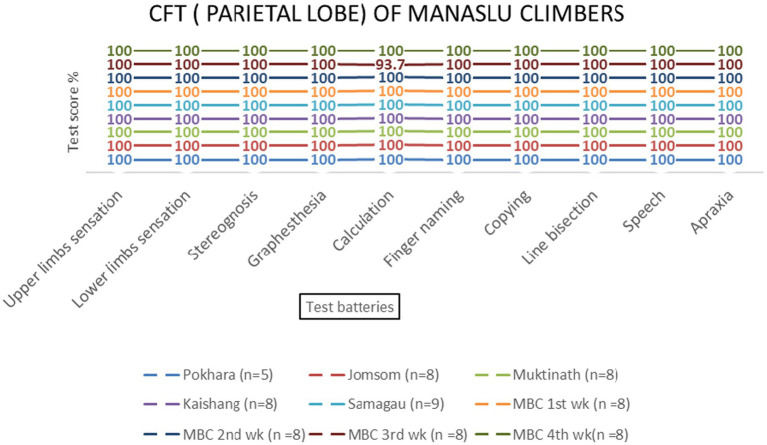
Parietal lobe examination scores of MMCL group across altitude stations (including once per week total four times at Manaslu Base Camp) based on 10 test batteries.

No significant effects of altitude were observed for parietal lobe functions [*F* (3, 69) = 1.23, *p* = 0.306]. All test batteries showed consistent performance across altitudes (Table 2 and Figures 6–9).

**Table 2:** ANOVA results for parietal, temporal, and occipital lobe functions.

Lobe	*F*-value	*p*-value	Partial *η*^2^	*Post-hoc* (Bonferroni)
Parietal	1.23	0.306	0.05	NS
Temporal	0.89	0.451	0.03	NS
Occipital	0.67	0.573	0.02	NS

### 3.3 Temporal and occipital lobe functions

**Figure 10:**
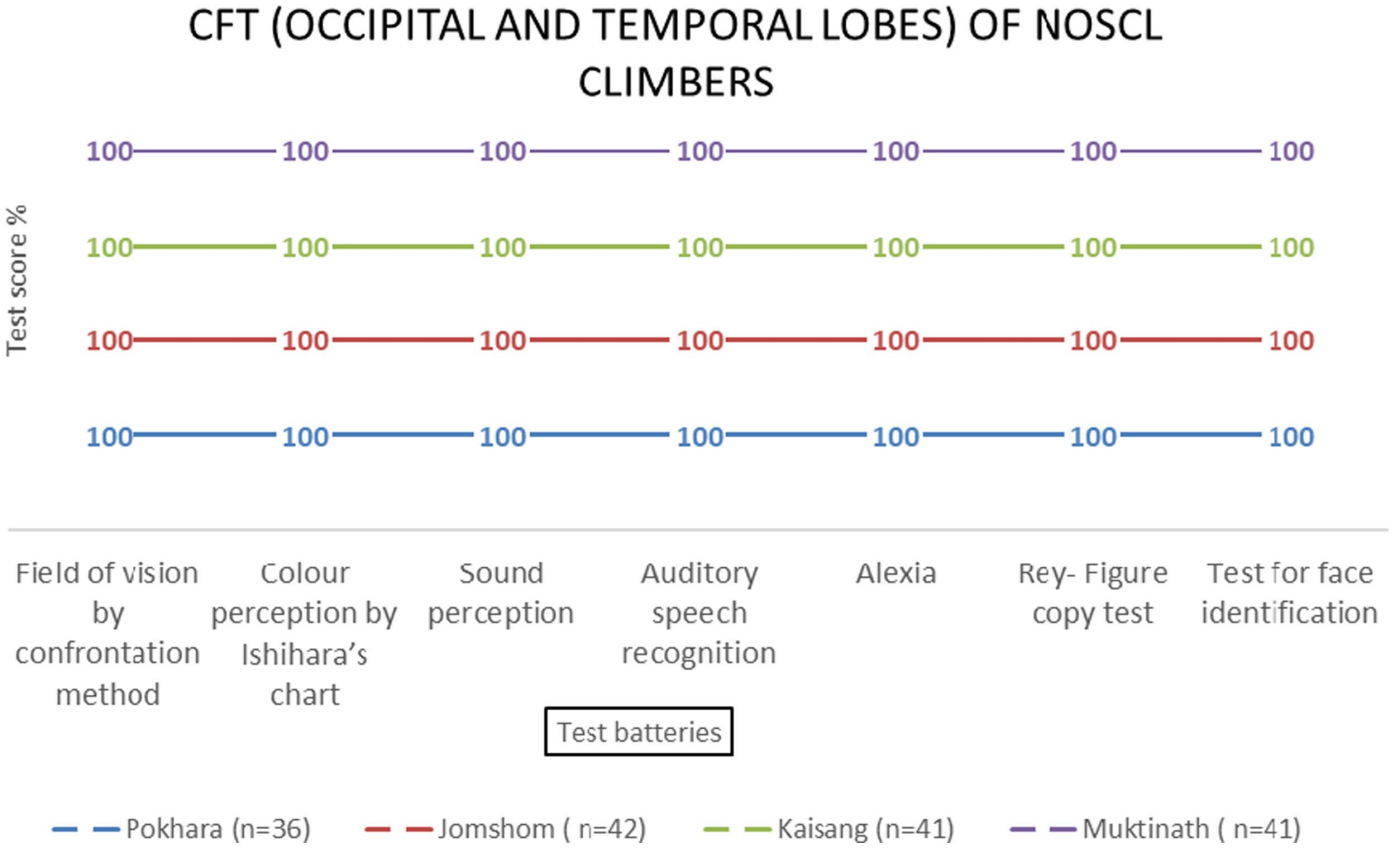
Occipital and temporal lobe examination scores of NOSCL group at four stations based on two (field of vision by confrontation method & color perception by Ishihara’s chart) and five test batteries, respectively.

**Figure 11:**
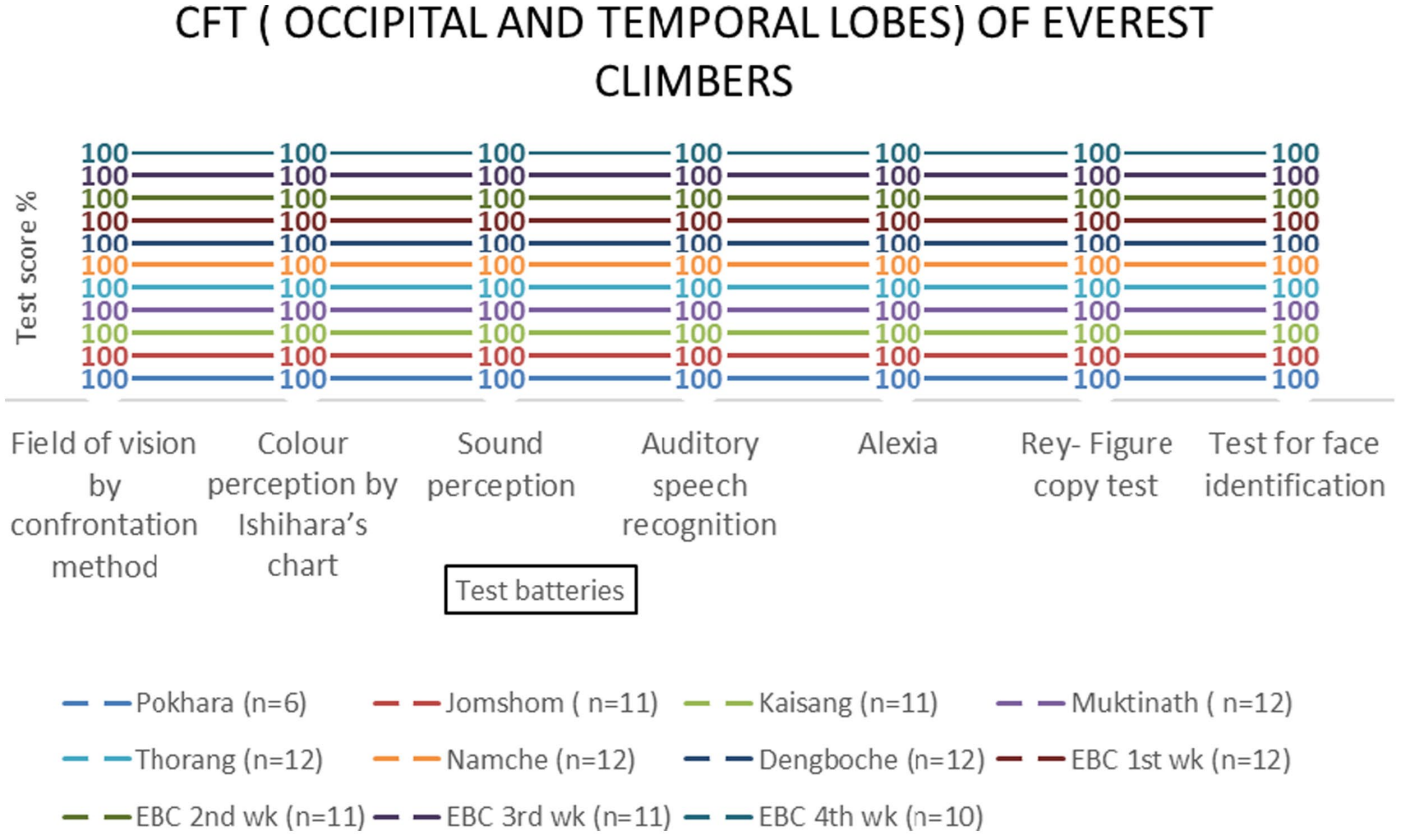
Occipital and temporal lobes examination scores of EMCL group across altitude stations (including once per week total four times at Everest Base Camp) based on two (field of vision by confrontation method & color perception by Ishihara’s chart) and five test batteries, respectively.

**Figure 12:**
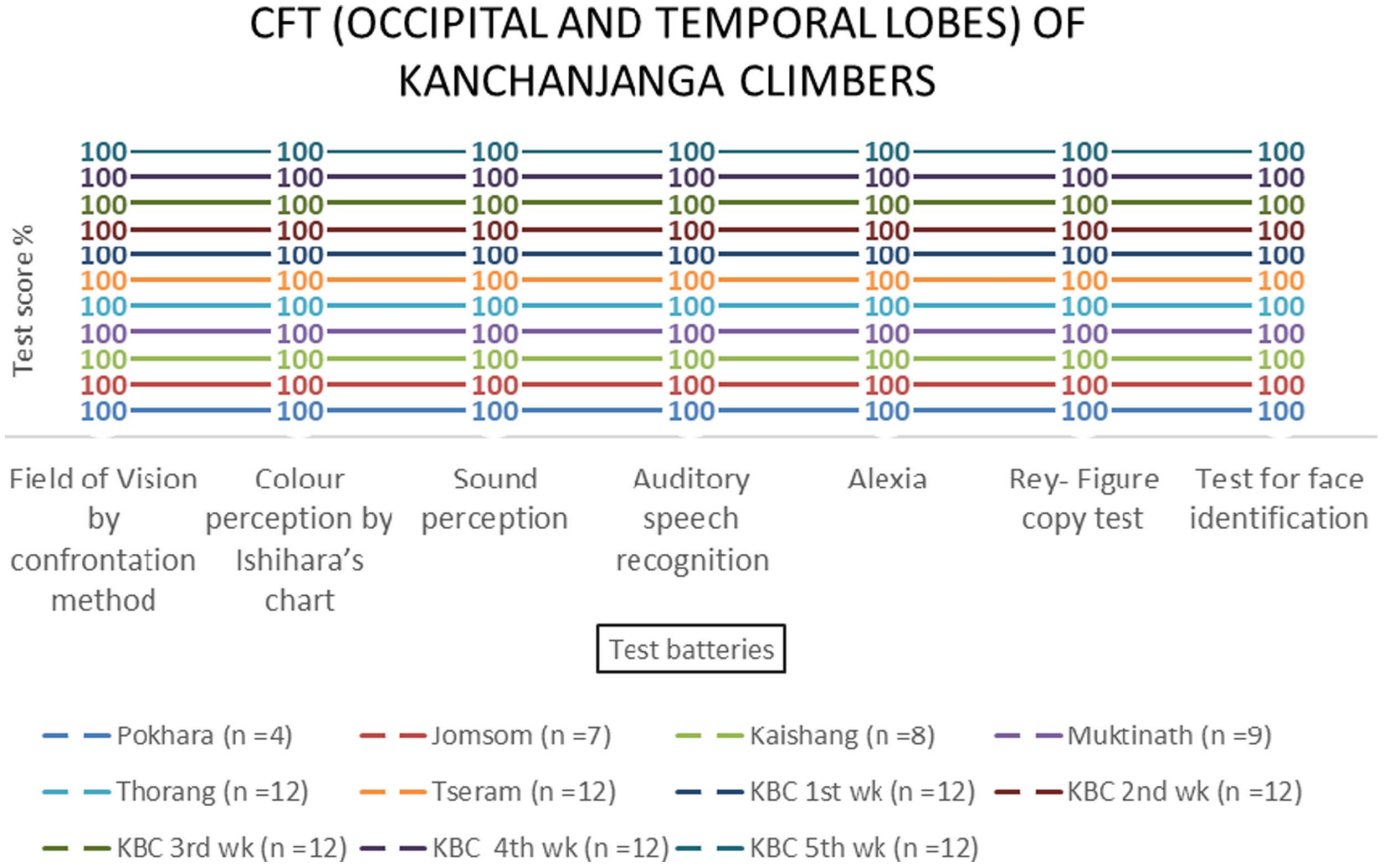
Occipital and temporal lobes examination scores of KMCL group across altitude stations (including once per week total four times at Kanchanjanga Base Camp) based on two (field of vision by confrontation method & color perception by Ishihara’s chart) and five test batteries, respectively.

**Figure 13:**
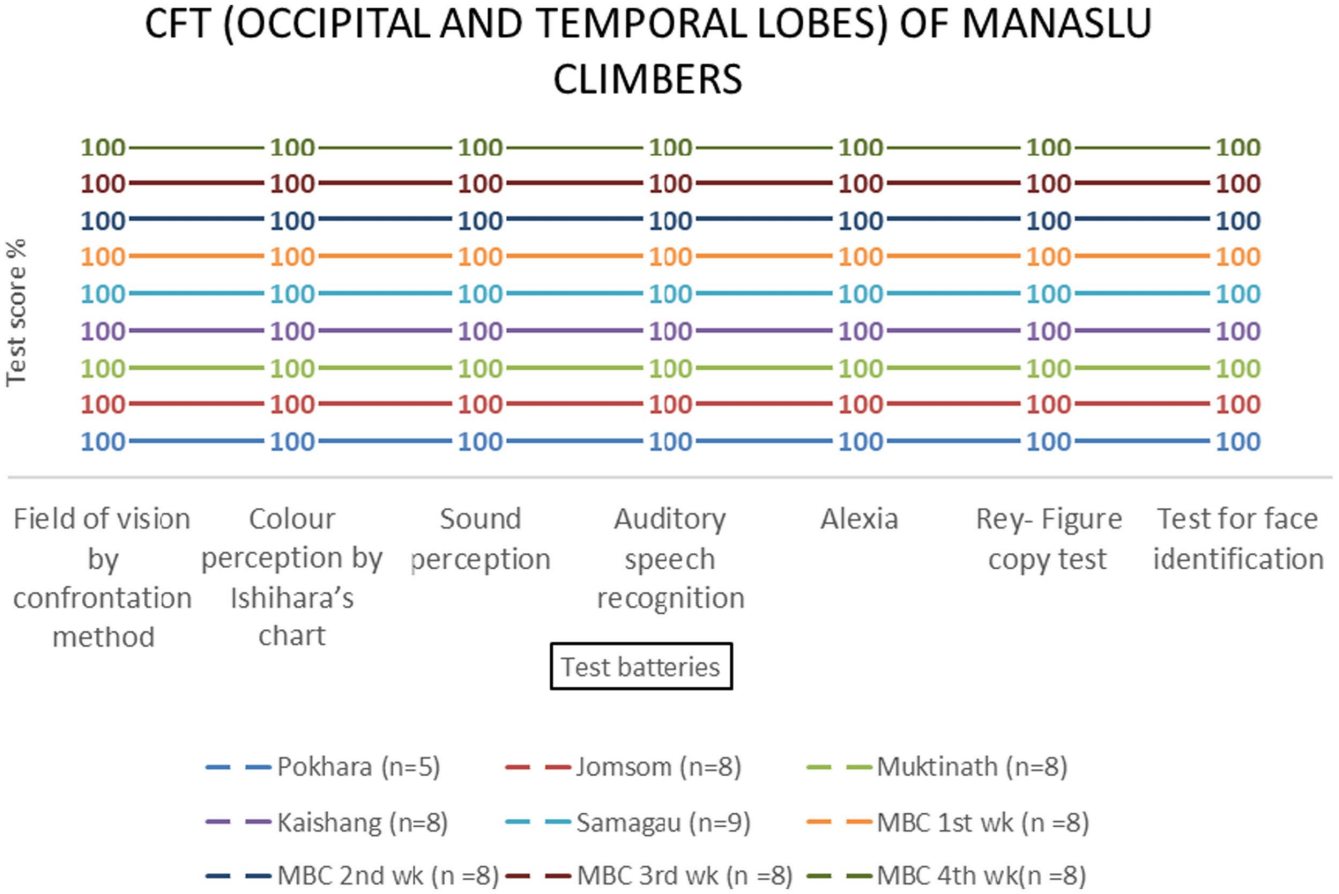
Occipital and temporal lobes examination scores of MMCL group across altitude stations (including once per week total four times at Manaslu Base Camp) based on two (field of vision by confrontation method & color perception by Ishihara’s chart) and five test batteries, respectively.

Temporal and occipital lobe functions remained unaffected by altitude [*F* (3, 69) = 0.89, *p* = 0.451 and *F* (3, 69) = 0.67, *p* = 0.573, respectively] (Table 2 and Figures 10–13).

### 3.4 Group comparisons

**Figure 14:**
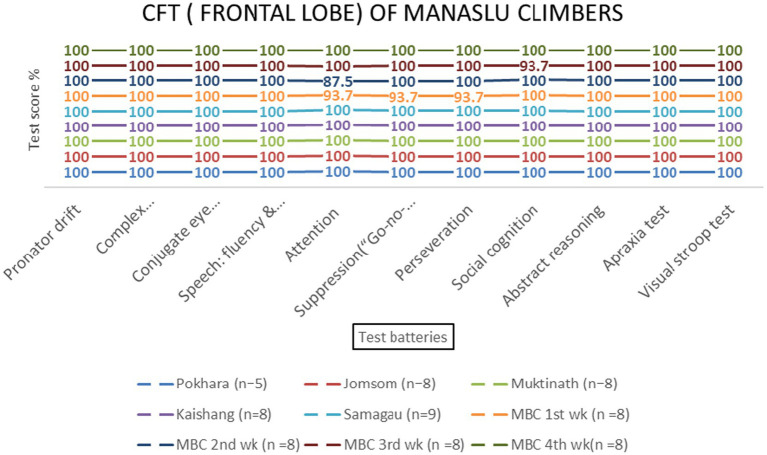
Frontal lobe examination scores of MMCL group across altitude stations (including once per week total four times at Manaslu Base Camp) based on 11 test batteries.

The Manaslu Mountain Climb (MMCL) group exhibited greater cognitive strain compared to the Everest (EMCL) and Kanchanjanga (KMCL) groups, particularly in attention and social cognition tasks at 4,800 meters (*p* = 0.028) (Figure 14).

## 4 Discussion

This study investigated the effects of high-altitude exposure on cerebral lobe functions in climbers, focusing on the frontal, parietal, temporal, and occipital lobes. The findings reveal significant altitude-related impairments in frontal lobe functions, particularly in attention and response suppression tasks, while parietal, temporal, and occipital lobe functions remained largely unaffected. These results align with and expand upon previous research on high-altitude cognitive impairments, offering new insights into the specific brain regions vulnerable to hypoxia (Kong et al., 2011; Falla et al., 2021; McMorris et al., 2017; Jung et al., 2020).

### 4.1 Frontal lobe vulnerability

The frontal lobe, responsible for executive functions such as attention, response suppression, and social cognition, was the most affected by high-altitude hypoxia. Visual Stroop test scores decreased significantly at 800 meters (75%, *p* < 0.001) and 2,700 meters (86.1%, *p* < 0.001), indicating executive control’s sensitivity to hypoxia at these altitudes. These findings are consistent with studies showing that hypoxia preferentially impairs higher-order cognitive functions mediated by the prefrontal cortex (Wilson et al., 2009; Yan, 2014). The observed decline in attention scores at 800 meters (94.4%, *p* = 0.002) and 4,800 meters (87.5%, *p* = 0.145) further supports the vulnerability of the frontal lobe to altitude-induced hypoxia. This aligns with research by Wang et al. (2014), who reported slower response times and altered hemispheric compensation in attention tasks at high altitudes.

### 4.2 Parietal, temporal, and occipital lobe resilience

In contrast to the frontal lobe, parietal, temporal, and occipital lobe functions showed no significant altitude-related impairments. The resilience of these regions may reflect their lower metabolic demands compared to the frontal lobe (Hochachka et al., 1994). For instance, the occipital lobe’s role in visual processing appears unaffected by hypoxia, as evidenced by unchanged scores in color perception and visual field tests. This finding aligns with studies reporting no significant changes in color vision at high altitudes (Leid and Campagne, 2001; Davies et al., 2011). Similarly, the temporal lobe’s auditory and memory functions remained intact, consistent with research showing that acclimatized climbers exhibit no significant deficits in auditory processing (Merz et al., 2013; Virues-Ortega et al., 2011).

### 4.3 Manaslu’s cognitive challenges

The Manaslu climb posed greater cognitive challenges compared to Everest and Kanchanjanga, as evidenced by decreased attention and social cognition scores at 4,800 meters (*p* = 0.028). This may reflect the unique environmental and psychological stressors associated with Manaslu, such as steeper ascents and prolonged exposure to extreme altitudes. These findings highlight the importance of mountain-specific acclimatization strategies and accentuate the need for further research on the interplay between environmental factors and cognitive performance.

### 4.4 Comparison with previous studies

The findings of this study are consistent with previous research demonstrating the frontal lobe’s sensitivity to hypoxia (Wilson et al., 2009; Yan, 2014). However, the lack of significant effects on parietal, temporal, and occipital lobe functions contrasts with some studies reporting altitude-related impairments in spatial awareness and vision (Jung et al., 2020; Yan et al., 2011). This discrepancy may stem from differences in acclimatization protocols or the use of non-invasive test batteries in the current study (Banasiewicz et al., 2014; Karakucuk and Mirza, 2000; Gao et al., 2015). Additionally, the observed resilience of the temporal lobe aligns with research showing no significant deficits in auditory processing among acclimatized climbers (Banasiewicz et al., 2014). The acclimatization period of 3–7 nights at each altitude station was based on standard protocols for stabilizing peripheral oxygen saturation (SpO₂) and reducing acute mountain sickness (AMS) risk (Bärtsch and Swenson, 2013; West et al., 2012). This duration aligns with evidence suggesting that most lowlanders achieve physiological stability within this timeframe, particularly at altitudes below 4,000 meters (Luks et al., 2017).

## 5 Implications and future directions

Future research should integrate physiological measures (e.g., cerebral oxygenation, biomarkers) with cognitive testing to elucidate mechanisms of altitude-related deficits. Longitudinal designs and diverse cohorts (e.g., females, ethnic groups) are needed.

### 5.1 Limitations

This study lacked physiological measures (e.g., SpO₂), had a male-dominated sample (89.4%), and used composite batteries that may confound specific cognitive domains.

## 6 Conclusion

Frontal lobe functions, particularly executive control, are highly vulnerable to high-altitude hypoxia, while other lobes remain resilient. These findings underscore the value of cerebral lobe-specific testing for climbers and military personnel. Future work should combine cognitive assessments with physiological monitoring to refine altitude acclimatization protocols.

## Data Availability

The raw data supporting the conclusions of this article will be made available by the authors, without undue reservation.
