# Supplementary material for: Impact of high-altitude exposure on cerebral lobe functions in climbers: insights from the Nepali Himalayas
Source: Front Syst Neurosci. 2025 May 30;19:1563398. doi: 10.3389/fnsys.2025.1563398 (PMC12162956; doi:10.3389/fnsys.2025.1563398)
Supplement: Supplementary file 1 [file Data_Sheet_1.pdf]

10. Pro forma (Annex - I) attached separately

Department of Clinical Physiology, NAIHS

Worksheet: Cerebral Lobe Function Tests for climbers

"Prevalence of cerebral lobe functions following acquaintance to high altitude of Nepali Himal" **Dhungel et al**

Part 1: Subject sign in. They are provided worksheets. Supervisor gives a brief instruction on lobe function tests using the worksheet (20 min duration).

Then distribute following worksheet to individual participant

Part 2: (30-45 minutes) / per subject one by one under the supervision of an assigned member of project and perform the following tests and note down their observations.

A. FRONTAL LOBE FUNCTION TESTS: for climber/ subject in different HA

**Test**	**Procedure** for subject/ climber	**Observation**
**1. Simple motor upper limbs** 1. Inspect for wasting in muscles<br>**2.** Tone<br>**3.** Shoulder abduction 4. Forearm extension and flexion 5.Grip<br>6. Finger abduction Opposition thumb<br>7. Pronator drift	Testing for pronator drift<br>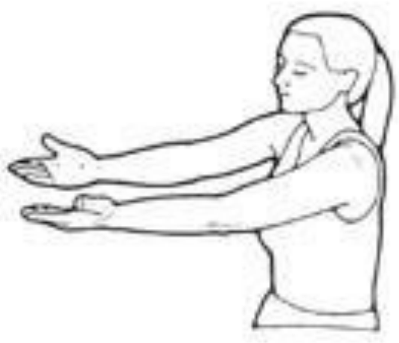<br>Subject holds hands supine<br>Eyes closed<br>50 seconds duration Normal response:<br>Hand remains supine.	Circle appropriately<br>0-1-2<br>(Hands remain supine=2, Prone =0, confuse/ not sure=1)
**2. Simple motor lower limbs** 1. Inspect for wasting in muscles<br>2. Tone<br>3. Hip flexion<br>4. Hip extension<br>5. Hip adduction<br>6. Hip abduction<br>7. Knee flexion<br>8. Knee extension<br>9. Ankle dorsiflexion 10. Ankle plantar flexion<br>11. Great toe dorsiflexion<br>12. Great toe plantar flexion.		

**3. Complex sequence**	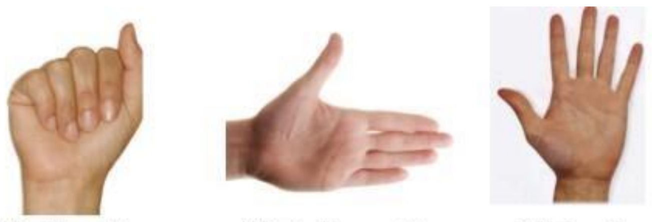<br>"Fist" "Edge" "Palm"<br>Subject makes sequence of movements:	Circle appropriately<br>0-1-2<br>Could make sequence =2, Could not make sequence =0; partial sequence=1)

**4. Conjugate eye-movements**	Subject moves the eyes in various directions:<br>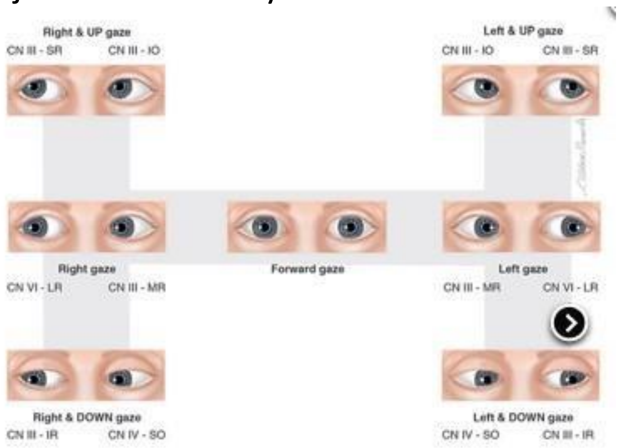	Circle appropriately<br>0-1-2
**5. Speech**<br>Fluency<br>Repetition	1. Repeat: *"I only knew John was the one to help today" "The cat always hid under the couch when the dogs were in the room"* *(आवश्यक परको बेलामा मलाइ सहयोग गर्ने राम मात्र हो भन्ने मलाइ थाहा थियो/जब कुकुर कोठामा आउछ तव बिरालो कुर्सी मुनि लुक्छ)*	Circle appropriately<br>0- 1- 2
**6. Attention**	2. Fluency: Name maximum number of words beginning with "F" in one minute. (No proper nouns)<br>"क" बर सुरु हुन शब्द हरु भन्नुस<br>Read list of digits- 1/sec.<br>तलका अंक हरु क्रिम मिलायर भन्नुस<br>अघाडी: २,१,८,५,४<br>पछाडी: ७,४,२	Circle appropriately<br>0- 1- 2<br>एक मिनट मा ६ वोटा बडी शब्द = २<br>६ कम = १<br>भन्न नसक्ने = ०
**7. Suppression "Go-no-go"**	Subject moves his finger up when he hears one tap on the table and keeps the finger raised when he hears two taps.<br>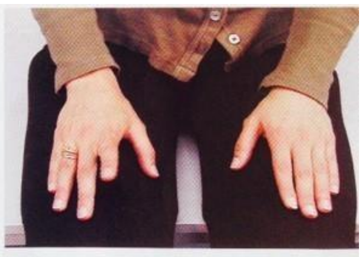<br>Auditory go–no–go<br>Explain procedure in Nepali	Circle appropriately<br>0-1-2

**8. Perseveration**	Subject needs to copy alternating sequence of triangle and squares drawn by examiner<br>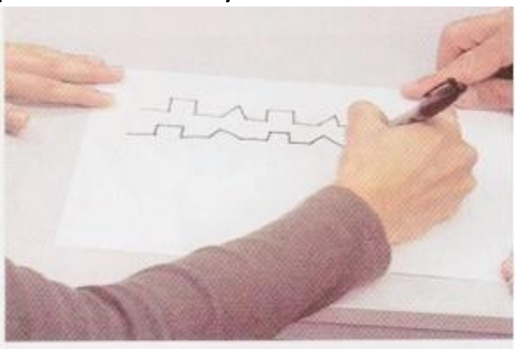<br>Written alternating sequencing test<br>Explain in Nepali	Circle appropriately 0-1-2
**9. Social cognition**	*Choose the facial expression from the list:*<br>*1. Happy 2. Sad 3. Angry 4. No expression 5. Scared*<br>• ***For the person on the right side in 'A'***<br>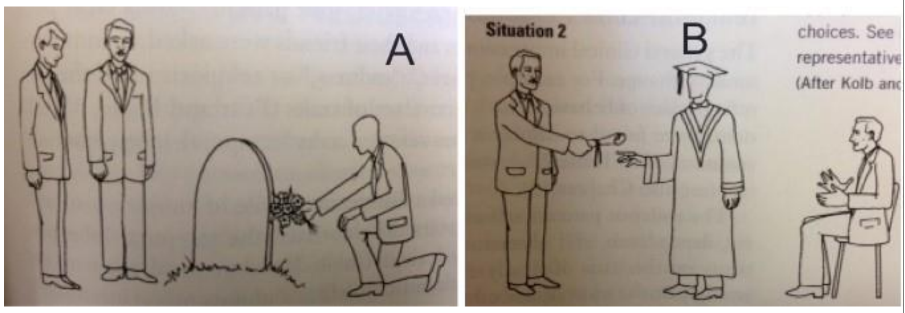<br><br>• ***For the person in the middle in 'B'***	Circle appropriately 0-1-2
**10. Abstract reasoning**	*What is the similarity between:*<br>*Car and bicycle*<br>*Banana and apple*<br>*Rose and Jasmine*<br>*Watch and ruler*<br>(We can ask in Nepali)	Circle appropriately 0-1-2
**11. Test for apraxia**	***Imitate the examiner***<br>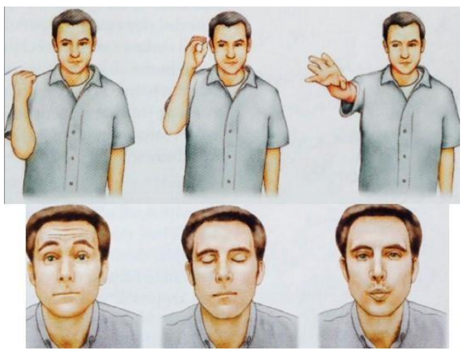<br>(We can ask in Nepali)	Circle appropriately 0-1-2

12. Suppression of inappropriate response: Visual Stroop test.
Subject needs to list the colors without reading them ***Circle appropriately 0-1-2***

अक्ष्यर नपदी कन रंग को नाम भन्नुस

निलो हरियो पहेलो गुलाबी बैजनी कालो खैरो रातो

BLUE GREEN YELLOW

PINK RED ORANGE

GREY BLACK PURPLE

B. PARIETAL LOBE FUNCTION TESTS:

Test	Procedure	Observation
**1. Simple sensations upper limbs**<br>Crude and fine touch<br>Two-point discrimination<br>Tactile localization<br>Tactile discrimination<br>Joint sensation<br>Vibration sensation	**Supervisor or assigned person need to perform**	Circle appropriately 0-1-2
**2. Simple sensations lower limbs**<br>Crude and fine touch<br>Two-point discrimination<br>Tactile localization<br>Tactile discrimination<br>Joint sensation<br>Vibration sensation	**Supervisor or assigned person need to perform**	Circle appropriately 0-1-2
**3. Stereognosis**<br><br>**4. Graphesthesia**	Subject recognizes common objects by feeling eyes closed.<br>Subject recognizes simple scripts written on the palm, eyes closed.	Circle appropriately 0-1-2
**5. Calculation**	Subtract<br>(20-15=)<br>(60-43=)	Circle appropriately 0-1-2
**6. Finger-naming**	*Touch the thumb of your left hand with the forefinger of your right hand.*	Circle appropriately 0-1-2
**7. Copying**	*Copy the following figures*<br>A 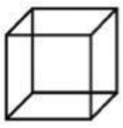<br>B<br>C 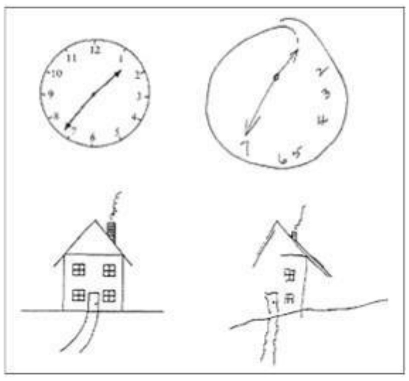	Circle appropriately 0-1-2

**8. Line bisection**	*Bisect the following lines:* तलका धर्का हरु लाई भिभाजन गर्नुस 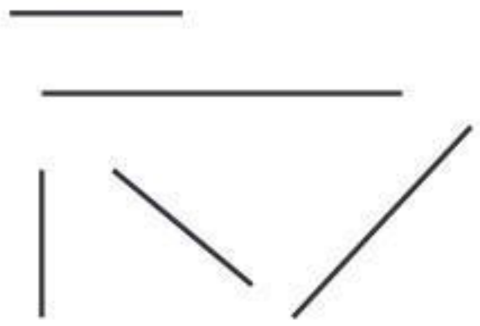	Circle appropriately 0-1-2
**Speech**	**(Tested with frontal lobe)**	
**Apraxia**	**(Tested with frontal lobe)**	

C. OCCIPITAL LOBE

**1. Field of vision by confrontation method**		Circle appropriately 0-1-2
**2. Colour perception by Ishihara's chart**	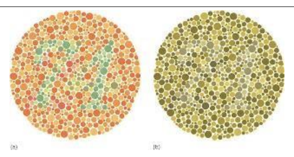	Circle appropriately 0-1-2

**3. Line drawings**	*Recognize the following line-drawings and copy them:* 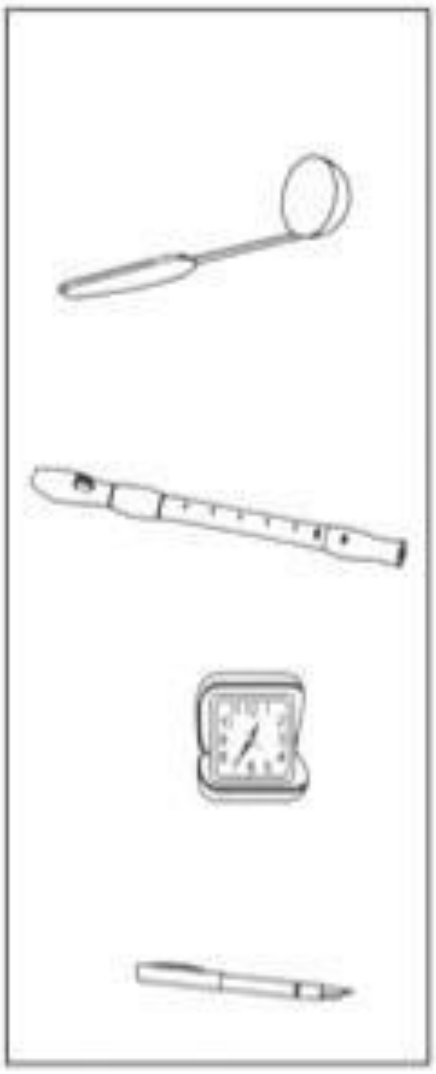	Circle appropriately 0-1-2

D. TEMPORAL LOBE

**1. Sound perception**	Ask the subject to	Circle appropriately 0-1-2
**2. Auditory speech recognition**	Subject responds verbally and in writing: *"What's your name?"* *"What kind of work do you do?"*	Circle appropriately 0-1-2
**3. Alexia**	Subject is asked to read what he/she wrote above	Circle appropriately 0-1-2
**4. Rey-Figure copy test**	Subject is asked to copy the figure: 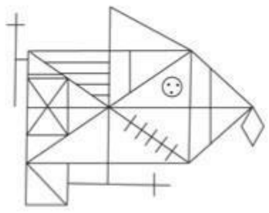 **Subject is asked to draw the figure by recall 20-minutes later:**	Circle appropriately 0-1-2
**5. Test for face identification**	*Identify the face in the figure below:* 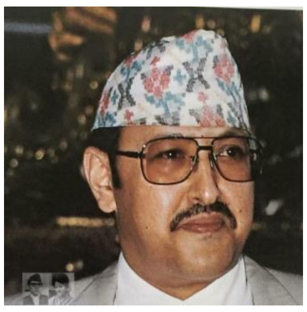	Circle appropriately 0-1-2

Need to prepare checklist by observer with their observation (i.e Circle appropriately 0-1-2 (0 wrong, 1, half correct, 2 correct)

References: Video link: Bedside frontal lobe tests: https://vimeo.com/15672155

Department of Clinical Physiology, NAIHS Letter of Written Consent:

“Prevalence of cerebral lobe functions following acquaintance to high altitude of Nepali Himal”

LETTER OF CONSENT

I Mr./Mrs……………………………………………..have been explained fully about the purpose and procedure of the study titled, “Prevalence of cerebral lobe functions following acquaintance to high altitude of Nepali Himal’’ that is going to be held in Shree Birendra hospital and Military Hospital Pokhara by Dr Shavana Rana. I am willingly participating in the study. I have been assured that confidentiality will be maintained to the utmost and no names, documents or results will be disclosed or circulated anywhere other than the hospital doctors or researchers. I am also aware that I have full rights to withdraw my participation from this study whenever I wish to do so. I will neither be charged extra costs nor be paid any extra benefits regarding this study. I am letting to do all examinations in and do all the investigations & procedures as required for the study. I hereby agree to participate in the study.

Participant Signature :

Consent taken by:

Left

Right

Full Name:

Finger Print
