# Supplementary material for: Impact of high-altitude exposure on cerebral lobe functions in climbers: insights from the Nepali Himalayas
Source: Front Syst Neurosci. 2025 May 30;19:1563398. doi: 10.3389/fnsys.2025.1563398 (PMC12162956; doi:10.3389/fnsys.2025.1563398)
Supplement: Supplementary file 2 [file Data_Sheet_2.pdf]

Cerebral functions test following acquaintance to high altitude of Nepal himal			
Name :			
Age:	Sex:		
Altitude &Place:			
Date of 1st test:			
Date of 2nd test:			
Date of 3rd test:			
Tests	Score		
Pronator drift	0	1	2
Complex sequence(Fist-Edge-Palm)	0	1	2
Conjugate eye movements	0	1	2
Speech: fluency & repetition	0	1	2
Attention	0	1	2
Suppression(“Go-no-go”)	0	1	2
Perseveration	0	1	2
Social cognition	0	1	2
Abstract reasoning	0	1	2
Apraxia test	0	1	2
Visual strops test	0	1	2
Simple sensation upper limbs	0	1	2
Simple sensation lower limbs	0	1	2
Stereognosis	0	1	2
Graphesthesia	0	1	2
Calculation	0	1	2
Finger naming	0	1	2
Copying	0	1	2
Line bisection	0	1	2
Speech	0	1	2
Apraxia	0	1	2
Field of vision by confrontation method	0	1	2
Colour perception by Ishihara’s chart	0	1	2
Sound perception	0	1	2
Auditory speech recognition	0	1	2
Alexia	0	1	2
Rey- Figure copy test	0	1	2
Test for face identification	0	1	2

Cerebral functions test following acquaintqnce to high altitude of Nepal himal			
Name :			
Age:	Sex:		
Altitude &Place:			
Date of 1st test:			
Date of 2nd test:			
Date of 3rd test:			
Tests	Score		
Pronator drift	0	1	2
Complex sequence(Fist-Edge-Palm)	0	1	2
Conjugate eye movements	0	1	2
Speech: fluency & repetition	0	1	2
Attention	0	1	2
Suppression(“Go-no-go”)	0	1	2
Perseveration	0	1	2
Social cognition	0	1	2
Abstract reasoning	0	1	2
Apraxia test	0	1	2
Visual strops test	0	1	2
Simple sensation upper limbs	0	1	2
Simple sensation lower limbs	0	1	2
Stereognosis	0	1	2
Graphesthesia	0	1	2
Calculation	0	1	2
Finger naming	0	1	2
Copying	0	1	2
Line bisection	0	1	2
Speech	0	1	2
Apraxia	0	1	2
Field of vision by confrontation method	0	1	2
Colour perception by Ishihara’s chart	0	1	2
Sound perception	0	1	2
Auditory speech recognition	0	1	2
Alexia	0	1	2
Rey- Figure copy test	0	1	2
Test for face identification	0	1	2
